# Therapeutic potential of hydroxybenzoic acid and lentinan in mitigating glucocorticoid-induced osteoporosis: an experimental study

**DOI:** 10.3389/fphar.2026.1957515

**Published:** 2026-10-26

**Authors:** Busra Soyler, Tugba Melike Parlak, Ibrahim Buyuktaskapulu, Muhammed Demircioglu, Muhammet Bahaeddin Dortbudak, Ali Kocyigit, Ismail Demircioglu, Annalisa Chiavaroli, Burak Dik

**Affiliations:** 1 Department of Pharmacology and Toxicology, Faculty of Veterinary Medicine, Selcuk University, Konya, Türkiye; 2 Department of Histology and Embryology, Faculty of Medicine, Istanbul Aydın University, Istanbul, Türkiye; 3 Department of Pathology, Faculty of Veterinary Medicine, Harran University, Sanliurfa, Türkiye; 4 Department of Anatomy, Faculty of Veterinary Medicine, Harran University, Sanliurfa, Türkiye; 5 Department of Pharmacy, Gabriele d’Annunzio University, Chieti, Italy

**Keywords:** glucocorticoids, hydroxybenzoic acids, lentinan, osteoporosis, RANK/RANKL pathway, TNF-α/IL-1β expression

## Abstract

**Introduction:**

The present study aimed to elucidate the therapeutic efficacy and mechanistic roles of Hydroxybenzoic Acid and Lentinan in counteracting glucocorticoid-induced osteoporosis (GIOP) in mice, with a focus on bone metabolism, inflammation, and oxidative stress pathways.

**Methods:**

Sixty-eight male Swiss Albino mice were randomly assigned to seven groups: Healthy Control, Osteoporosis (methylprednisolone 10 mg/kg), Teriparatide (3 μg/kg), Low-Dose Lentinan (200 mg/kg), High-Dose Lentinan (400 mg/kg), Low-Dose HBA (50 μg/kg), and High-Dose HBA (100 μg/kg). In the study, all treatments were administered daily for a duration of 10 weeks.

**Results and Discussion:**

Methylprednisolone administration resulted in marked deterioration of bone quality, with significant reductions in 25(OH)D3, IGF-1, and increased osteocalcin, TNF-α, IL-1β, HSP-27, RANK, and RANKL expression, accompanied by severe trabecular bone loss, all treatment groups showed notable recovery, with the most pronounced restoration in teriparatide and high-dose hydroxybenzoic acid groups. Mechanistically, hydroxybenzoic acid exerted potent anti-osteoclastic effects through RANK/RANKL pathway suppression, while Lentinan enhanced osteoblastic activity and mitigated inflammatory damage. In conclusion, these results provide preclinical evidence that HBA and lentinan warrant further investigation as potential adjuncts to established anti-osteoporotic therapy in the setting of long-term glucocorticoid use.

## 1 Introduction

Osteoporosis is a chronic skeletal disorder characterized by reduced bone strength and an increased risk of fragility fractures (Giangregorio et al., 2009). Bone remodeling is regulated by complex molecular pathways involving receptor activator of nuclear factor-kappa B (RANK), its ligand RANKL, and the decoy receptor osteoprotegerin (OPG). RANKL, secreted primarily by osteoblasts, binds to RANK on osteoclast precursors, promoting their activation and bone resorption, whereas OPG binds RANKL and inhibits osteoclastogenesis (Blair et al., 2007; Theoleyre et al., 2004). Osteoporosis is classified as primary or secondary. Primary osteoporosis includes postmenopausal (Type I) and age-related (Type II) forms, whereas secondary osteoporosis results from underlying diseases or long-term pharmacological interventions, particularly glucocorticoid (GC) therapy, a major cause of drug-induced osteoporosis (Cosman et al., 2014; Hannan et al., 2000).

Glucocorticoids are widely used to treat chronic inflammatory and autoimmune conditions; however, prolonged administration, particularly at doses equivalent to ≥5 mg/day of prednisolone for more than 3 months, is strongly associated with bone loss and increased fracture risk (Compston, 2010; Hartmann et al., 2016). The pathogenesis of glucocorticoid-induced osteoporosis (GIOP) is multifactorial and biphasic, involving an initial increase in bone resorption followed by impaired bone formation. GCs reduce intestinal calcium absorption, increase renal calcium excretion, suppress gonadotropin and sex steroid production, and inhibit osteogenic factors such as IGF-1 and TGF-β. They also induce osteoblast and osteocyte apoptosis, impair VEGF-mediated vascularization, and alter the RANK/RANKL/OPG axis in favor of osteoclastogenesis (Ilias et al., 2022; Liu et al., 2013). In addition, persistent pro-inflammatory signaling involving TNF-α, IL-1, and IL-6 may further contribute to bone degradation (Hartmann et al., 2016; Ritchlin et al., 2003). Overall, GCs promote osteoporosis through mechanisms that enhance bone resorption and suppress bone formation (Hartmann et al., 2016).

Benzoic acid and its derivatives are phenolic compounds with reported effects on bone metabolism, particularly through modulation of oxidative stress and inflammation. Hydroxybenzoic acid derivatives have been shown to inhibit RANKL-induced osteoclast differentiation by reducing reactive oxygen species (ROS) production, thereby attenuating bone resorption (Zhang et al., 2024). Natural phenolic compounds may also enhance osteoblast activity while suppressing osteoclast function, potentially shifting bone remodeling toward bone formation (Jiao et al., 2009).

Among these compounds, p-hydroxybenzoic acid (4-hydroxybenzoic acid; 4-HBA) and its sodium salt, sodium p-hydroxybenzoate (DSN160), have attracted particular interest because of their reported antioxidant, anti-inflammatory, and estrogen-like properties (Manuja et al., 2013; Safarova, 2022; Wang et al., 2020; Lemini et al., 2003; Lemini et al., 1997). p-Hydroxybenzoic acid is naturally present in edible plants and fungi, including *Daucus carota* and *Vitis vinifera*, and exhibits antimicrobial, anti-inflammatory, antioxidant, and estrogen-like activities (Manuja et al., 2013; Safarova, 2022; Wang et al., 2020). Previous studies have reported that p-hydroxybenzoic acid can interact with estrogen receptors (ERs) and modulate ER expression, suggesting a potential contribution of estrogen-related signaling to its bone-protective effects (Lemini et al., 2003; Lemini et al., 1997). Consistent with this possibility, DSN160 improved bone mass and trabecular microarchitecture in retinoic acid–induced osteoporosis models, while these effects were abolished by ER antagonism, indicating an important role for ERα signaling in addition to the attenuation of oxidative stress (Xu et al., 2023).

Furthermore, methyl 3,4-dihydroxybenzoate has been shown to enhance antioxidant defense through Nrf2 signaling, inhibit osteoclastogenesis, and reduce bone loss in LPS-induced osteolysis and ovariectomy-induced osteoporosis models (Huang et al., 2022). Hydroxybenzoic acids may also suppress pro-inflammatory cytokines such as TNF-α and IL-6 through modulation of NF-κB and MAPK signaling, thereby reducing bone resorption (Gong and Zha, 2023; Su et al., 2024). Comparative studies suggest that DSN160 acts predominantly through ERα-dependent signaling, whereas related benzoic acid derivatives may exert anti-osteoporotic effects through osteoclastogenic and antioxidant pathways (Huang et al., 2022; Jang et al., 2018). Collectively, these findings support the potential of benzoic acid derivatives to preserve bone health through antioxidant, anti-inflammatory, estrogen-like, and osteoclast-related mechanisms (Wang et al., 2020; Su et al., 2024).

Lentinan (LNT), a β-(1,3)-D-glucan derived from *Lentinula edodes*, is a well-characterized immunomodulatory polysaccharide with established anti-inflammatory properties and has been clinically employed as an adjuvant therapeutic agent in cancer treatment in Japan and China (Lee H. J. et al., 2020; Saif et al., 2013). Although direct evidence for an anti-osteoporotic effect of lentinan in terms of bone mineral density is limited, mechanistic studies suggest potential benefits for bone–joint health. In human chondrocyte-like cells, lentinan suppresses AGE-induced inflammation through inhibition of NF-κB and p38 MAPK signaling, reducing nitric oxide, prostaglandin E_2_, and matrix-degrading enzymes including MMP-1, MMP-3, MMP-13, ADAMTS-4, and ADAMTS-5 (Zhang et al., 2020). In rat osteoarthritis models, lentinan also promotes chondrocyte mitophagy through the mTOR/PINK1/Parkin pathway, preserves extracellular matrix integrity, and attenuates cartilage degeneration (Li et al., 2025). Thus, lentinan demonstrates chondroprotective and anti-inflammatory effects that may indirectly support skeletal integrity under inflammatory or degenerative conditions, although its direct role in osteoporosis prevention remains to be established (Li et al., 2025; Erjavec et al., 2016; Parola et al., 2017).

Glucocorticoids, although essential in managing chronic inflammatory conditions, are known to induce secondary osteoporosis, especially in young individuals, due to suppressed bone formation and enhanced resorption. The adverse skeletal effects of glucocorticoids necessitate the development of supportive therapeutic strategies. In this study, we hypothesize that *p*-hydroxybenzoic acid, owing to its estrogenic and antioxidant profile, and lentinan, due to its immunomodulatory and osteogenic activities may protect against glucocorticoid-induced osteoporosis. Accordingly, this study is designed to comprehensively elucidate the anti-osteoporotic potential of both compounds in a well-established mice model of glucocorticoid-induced osteoporosis, through an integrative assessment encompassing biochemical markers, detailed histopathological evaluation, and targeted immunohistochemical analyses.

## 2 Materials and methods

### 2.1 Animal material and experimental design

A total of 68 male Swiss albino mice aged 8–10 weeks were used in this study. Prior to the experiment, all animals underwent health screening. Throughout the trial period, animals were housed under standard laboratory conditions with *ad libitum* access to food and water. The composition of the basal diet was as follows: dry matter 89%, crude protein 21%, crude fiber ≤5%, crude ash ≤10%, calcium 1%–2%, phosphorus 0.5%–1%, sodium chloride 0.5%, and metabolizable energy 2850 kcal/kg. The animals were maintained under controlled environmental conditions, with a 12-h light/12-h dark cycle (07:30–19:30), an ambient temperature of 22 °C ± 2 °C, and a relative humidity of 55% ± 5%. The mice were housed and maintained in polypropylene cages specifically designed and appropriate for laboratory mice throughout the experimental period.

The animals were randomly divided into seven experimental groups as outlined below. The induction of glucocorticoid-induced osteoporosis was performed based on the methodology described by Adhikary et al. (2019):

Group 1—Healthy Control (n = 8): Animals received 0.1 mL/kg/day isotonic saline via both oral and subcutaneous routes.
Group 2—Methylprednisolone (n = 10): Animals received 10 mg/kg/day subcutaneous methylprednisolone (Prednol-L® 40 MG IM/IV, Gensenta İlaç Sanayi ve Ticaret A.Ş., Turkiye) to induce osteoporotic changes.
Group 3—Methylprednisolone + Low Dose p-Hydroxybenzoic Acid (n = 10): Animals received 10 mg/kg/day methylprednisolone and 50 μg/kg/day subcutaneous 4-hydroxybenzoic acid (4-Hydroxybenzoic acid, HY-Y0264, MedChemExpress, United States) (Lemini et al., 1997).
Group 4—Methylprednisolone + High Dose p-Hydroxybenzoic Acid (n = 10): Animals received 10 mg/kg/day methylprednisolone and 100 μg/kg/day subcutaneous 4-hydroxybenzoic acid (4-Hydroxybenzoic acid, HY-Y0264, MedChemExpress, United States) (Lemini et al., 1997).
Group 5—Methylprednisolone + Low Dose Lentinan (n = 10): Animals received 10 mg/kg/day methylprednisolone and 200 mg/kg/day oral lentinan (Lentinan, sc-499200Ai Santa Cruz, United States) by gastric gavage (Lee H. J. et al., 2020).
Group 6—Methylprednisolone + High Dose Lentinan (n = 10): Animals received 10 mg/kg/day methylprednisolone and 400 mg/kg/day oral lentinan (Lentinan, sc-499200Ai Santa Cruz, United States) by gastric gavage (Lee H. J. et al., 2020).
Group 7—Methylprednisolone + Teriparatide (n = 10): Animals received 10 mg/kg/day methylprednisolone and 3 μg/kg/day subcutaneous teriparatide (Forsteo® 20 MCG/80 MCL, Inj., 2.4 ML, Lilly France S.A.S. France). (Yu and Su, 2020).

All animals were inspected daily for general health, posture, coat condition, activity and any sign of pain or distress, and body weight was recorded weekly throughout the 10-week period. Predefined humane endpoints, established before the start of the study, comprised a loss of more than 20% of the maximum recorded body weight, persistent inactivity or hunched posture, inability to reach food or water, or any other sign of severe or unrelieved pain or distress; an animal meeting any of these criteria would have been euthanised immediately and excluded from further analysis. No animal reached a humane endpoint, no unscheduled death occurred, and all 68 animals completed the study and were included in the analyses.

At the end of the 10-week treatment period, animals were anesthetized with subcutaneous injections of 5 mg/kg xylazine and 95 mg/kg ketamine. Blood samples were collected via cardiac puncture into EDTA-coated tubes and clot-activator serum tubes. Following blood collection, the animals were euthanized by decapitation, and femur bones were harvested and fixed in 10% neutral-buffered formalin.

All doses were selected on the basis of previously published *in-vivo* studies and were verified by allometric scaling to human-equivalent doses (HED) using the body-surface-area method (HED = animal dose/12.33 for mice) (Nair and Jacob, 2016). Methylprednisolone was administered at 10 mg/kg/day s.c. following the murine glucocorticoid-induced osteoporosis model of Adhikary et al. (2019), in which this regimen reproducibly induces trabecular bone loss within a feasible experimental period; rodents are considerably less sensitive than humans to the skeletal effects of glucocorticoids and therefore require supraphysiological exposure to develop the phenotype. This dose corresponds to an HED of approximately 0.81 mg/kg, i.e., about 49 mg/day of methylprednisolone (≈61 mg/day prednisolone equivalent) in a 60-kg adult, which lies within the range of high-dose glucocorticoid therapy used clinically in autoimmune and inflammatory disease and well above the ≥5 mg/day prednisolone threshold associated with glucocorticoid-induced bone loss. The model therefore reproduces severe rather than low-dose glucocorticoid exposure. Teriparatide was given at 3 μg/kg/day s.c. (Yu and Su, 2020), corresponding to an HED of approximately 14.6 µg/day in a 60-kg adult and thus closely approximating the clinically approved dose of 20 µg/day; it was included as a positive anabolic comparator. The doses of p-hydroxybenzoic acid (p-HBA) were selected based on previous *in vivo* evidence demonstrating its biological activity within the microgram dose range. Accordingly, p-HBA was administered at 50 and 100 μg/kg/day via subcutaneous injection, as these doses were previously shown to exert biological effects *in vivo* (Lemini et al., 1997). Lentinan (LNT) was administered orally by gavage at doses of 200 and 400 mg/kg/day, based on previous findings demonstrating therapeutic efficacy at these doses without evidence of systemic toxicity (Lee H. J. et al., 2020). These dose ranges were therefore selected to ensure biologically relevant and well-tolerated exposure while allowing the assessment of potential dose-dependent effects.

### 2.2 Hematological and biochemical analysis

Hematological parameters including white blood cell (WBC), red blood cell (RBC), hematocrit, and platelet (PLT) counts were measured from the EDTA samples using an automated hematology analyzer (Mindray Bio-Medical Electronics, Shenzhen, China). Serum samples were separated by centrifugation at 4,000 rpm and aliquoted into microtubes. Samples were pre-frozen at −20 °C for 12 h and stored at −80 °C until analysis.

Serum samples were thawed to room temperature before analysis. Routine biochemical parameters including alanine aminotransferase (ALT), aspartate aminotransferase (AST), glucose, blood urea nitrogen (BUN), creatinine, and total protein were measured using a BT-3000 plus autoanalyzer (Biotecnica Instruments, Rome, Italy). In addition to these routine parameters, specific biomarkers associated with osteoporosis were assessed using commercially available mouse-specific ELISA kits (BT Lab, China), following the manufacturer’s protocols. The evaluated biomarkers included osteocalcin (E0032Mo), bone-specific alkaline phosphatase (BALP; E0306Mo), type I collagen C-terminal telopeptide (CTX-1; E0184Mo), insulin-like growth factor 1 (IGF-1; E0037Mo), tartrate-resistant acid phosphatase 5b (TRACP-5b; E0314Mo), procollagen type I N-terminal propeptide (PINP; E2686Mo), and 25-hydroxyvitamin D3 (25-OH Vit D3; EA0066Mo). All absorbance readings were recorded using a microplate reader (MWGt Lambda Scan 200, Bio-Tek Instruments Inc).

### 2.3 Decalcification, histopathological, and immunohistochemical analyses

Prior to evaluation, all slides were coded by a researcher who took no part in the histological assessment, and the code was not broken until scoring had been completed; both examining pathologists were therefore blinded to group allocation. For each animal, 5 non-serial sections were examined, and 5 randomly selected fields per section were evaluated at ×200 magnification for both haematoxylin–eosin and immunohistochemical preparations. Each parameter was scored independently by two observers using the scales described above. Inter-observer agreement was assessed by weighted kappa statistics, and any discordant score was resolved by joint re-examination of the slide on a multi-head microscope; the consensus score was used in all subsequent analyses. For immunohistochemistry, sections in which the primary antibody had been omitted served as negative controls and were included in each staining run.

Following necropsy, the femoral bones were fixed in 10% neutral-buffered formalin for 24 h. Subsequently, the trimmed bone tissues were transferred to Morse solution (a mixture of 10% formalin and 20% formic acid) for decalcification. During the initial 3 days, the decalcification solution was refreshed every 12 h and then every 24 h for the remaining period. The progress of decalcification was monitored using a syringe needle, and the process was estimated to last approximately 2 weeks. Once decalcification was deemed sufficient, the specimens were rinsed under running tap water for 24 h.

The fixed, trimmed, and decalcified tissues were subjected to routine histological processing. This included dehydration through a graded ethanol series, clearing in xylene, and paraffin embedding. All steps were performed using a semi-automated open-system tissue processor. The processed tissues were embedded into paraffin blocks using a paraffin dispenser. Serial sections of 4 μm thickness were obtained from each block using a rotary microtome. The sections were mounted on standard glass slides for histopathological evaluation and on adhesive-coated slides for immunohistochemical analysis (Gorodetskaya and Markevich, 2022).

For histopathological examination, the bone sections were stained with hematoxylin and eosin (H&E). Slides were first incubated at 60 °C for approximately 1 hour to melt the paraffin, followed by deparaffinization in xylene and rehydration through descending ethanol concentrations. After rinsing in distilled water, sections were stained with hematoxylin, differentiated under running tap water, counterstained with eosin, dehydrated again in ascending ethanol series, and cleared in xylene. Coverslips were mounted using Entellan. Histopathological evaluation was performed under a light microscope, and findings such as fibrous tissue proliferation, osteoblast activity, osteoid matrix calcification, inflammatory infiltration, and vascular changes were scored semiquantitatively on a scale from 0 (none) to 3 (severe) (Gorodetskaya and Markevich, 2022).

For immunohistochemistry, adhesive-mounted bone sections were also incubated at 60 °C, followed by deparaffinization and rehydration. Endogenous peroxidase activity was blocked using 3% hydrogen peroxide (H_2_O_2_), followed by phosphate-buffered saline (PBS) washes. Antigen retrieval was performed using a heat-mediated method with three cycles of heating and cooling, followed by PBS rinsing. The staining area was delineated using a PAP pen, and non-specific binding was blocked with a protein blocking solution.

Primary antibodies were applied directly without an intermediate washing step and incubated overnight at +4 °C. The antibodies selected for this study targeted key markers relevant to bone metabolism and inflammation: RANK (Santa Cruz Biotechnology, United States, sc-52951, 1/100) RANKL (Santa Cruz Biotechnology, United States, sc-52950, 1/100), was employed to evaluate osteoclast activity; HSP27 (Santa Cruz Biotechnology, United States, sc-13132, 1/100) served as a marker of oxidative stress; and TNF-α (Santa Cruz Biotechnology, United States, sc-52746, 1/100) along with IL-1β (Santa Cruz Biotechnology, United States, sc-32294, 1/100) were used to assess the inflammatory response.

Following overnight incubation, the sections were washed with phosphate-buffered saline (PBS) and incubated with biotinylated secondary antibodies. This step was followed by the application of a streptavidin-peroxidase conjugate to amplify the signal. To visualize the antigen-antibody complexes, 3,3′-diaminobenzidine (DAB) was used as a chromogenic substrate. After achieving the desired staining intensity, sections were rinsed with distilled water and counterstained with Mayer’s hematoxylin to enhance nuclear contrast. The slides were dehydrated using an ascending alcohol series, cleared in xylene, and mounted with Entellan. The immunohistochemical staining was examined under a light microscope, and immunopositivity was scored semi-quantitatively based on staining intensity using a four-point scale: 0 (none), 1 (mild), 2 (moderate), and 3 (strong), in accordance with the method described by Hyväri et al. (2023).

### 2.4 Statistical analysis

All quantitative data obtained in the study were analyzed using the SPSS version 25.0 statistical software package (SPSS Inc., Chicago, IL, United States). Prior to analysis, the distribution characteristics of the data were assessed using the Shapiro-Wilk normality test, and the homogeneity of variances was evaluated with Levene’s test. For datasets that met the assumptions of normality and homogeneity of variance, parametric methods were employed. Specifically, group comparisons were conducted using one-way analysis of variance (One-Way ANOVA), and statistically significant differences were further analyzed through Tukey’s honestly significant difference (HSD) *post hoc* test for pairwise comparisons.

In cases where data did not follow a normal distribution or variances were not homogeneous, non-parametric methods were applied. The Kruskal–Wallis test was used to evaluate differences among groups, and when significant differences were observed, the Dunn-Bonferroni *post hoc* test was performed for multiple comparisons. A *p*-value of less than 0.05 was considered statistically significant in all analyses.

## 3 Results

### 3.1 Biochemical and bone turnover parameters

The present study observed significant alterations in biochemical parameters in mice with experimentally induced osteoporosis (Table 1). Regarding biochemical parameters, serum 25(OH)D_3_ and IGF-1 levels were significantly reduced in the osteoporosis group compared to the control group (*p* < 0.05). The 25(OH)D_3_ levels were significantly increased in the high-dose lentinan, low-dose HBA, and high-dose HBA groups compared to the osteoporosis group (*p* < 0.05). The IGF-1 level in the high-dose HBA group was comparable to that of the control group (*p* > 0.05). Osteocalcin levels were significantly elevated in the osteoporosis group compared to the control group (*p* < 0.05), whereas in the high-dose lentinan and high-dose HBA groups, osteocalcin levels were similar to those of the healthy control group (*p* > 0.05). In addition, creatinine, glucose, and phosphorus levels were significantly lower in the osteoporosis group compared to the control group (*p* < 0.05).

**TABLE 1:** Effects of hydroxybenzoic acid (50–100 μg/kg) and Lentinan (200–400 mg/kg) Administration on biochemical parameters in mice with an experimental osteoporosis model (mean ± SEM/median (IQR)).

Parameters/groups	Control	Osteoporosis	Teriparatide	Low-dose lentinan	High-dose lentinan	Low-dose HBA	High-dose HBA
25-Hydroxyvitamin D3 (µg/L)	52.63 ± 2.77	15.74 ± 1.58^#^	46.73 ± 1.87*	21.12 ± 1.29^#^	30.65 ± 2.89^#^ ^,^ *	26.53 ± 1.92^#^ ^,^ *	35.49 ± 1.89^#^ ^,^ *
Insulin-like growth factor (ng/mL)	4.54 (3.85)	2.25 (0.57)^#^	3.73 (2.59)*	2.71 (1.07)^#^	2.65 (1.68)^#^	3.06 (0.86)^#^	3.01 (0.89)
Osteocalcin (ng/mL)	0.08 (0.04)	0.50 (0.05)^#^	0.34 (0.10)	0.51 (0.07)^#^	0.48 (0.23)	0.59 (0.18)^#^	0.29 (0.09)
ALT median (U/L)	423.0 (4.50)	389.0 (157.75)	1341.0 (3209.0)	307.0 (265.0)	564.0 (164.0)	352.0 (205.0)	369.0 (174.0)
AST median (U/L)	579.0 (3.0)	462.50 (424.0)	4001.0 (3753.2)	785.0 (514.0)	1017.0 (131.0)	950.0 (593.50)	487.0 (549.50)
Calcium (mg/dL)	9.35 ± 0.04	8.90 ± 0.16	8.90 ± 0.24	9.15 ± 0.20	9.37 ± 0.12	9.24 ± 0.10	9.27 ± 0.15
Creatinine (mg/dL)	0.36 (0.02)	0.31 (0.01)^#^	0.33 (0.04)	0.31 (0.01)^#^	0.31 (0.02)^#^	0.32 (0.01)	0.30 (0.02)^#^
Phosphorus (mg/dL)	12.0 ± 0.07	9.38 ± 0.33^#^	11.84 ± 0.85*	9.18 ± 0.24^#^	11.90 ± 0.33*	13.04 ± 0.63*	10.52 ± 0.40
Blood urea nitrogen (BUN) (mg/dL)	29.0 (0.0)	25.50 (4.0)	16.50 (4.0)^#^ ^,^ *	17.0 (1.0)^#^ ^,^ *	20.0 (3.0)^#^ ^,^ *	24.0 (0.50)	25.0 (2.0)

* Indicates significant differences compared to the Osteoporosis group.

^#^ Indicates significant differences compared to the Healthy Control group.

Data for CTX-1 and BALP are presented in Figure 1, while data for PINP and TRACP-5b are shown in Figure 2. No statistically significant differences were observed among the groups for CTX-1 levels (*p* > 0.05). The PINP level in the osteoporosis group was significantly higher than that in the control group (*p* < 0.05). All treatment groups exhibited significantly lower PINP levels compared to the osteoporosis group (*p* < 0.05). TRACP-5b levels were significantly elevated in the osteoporosis group relative to the control group, and this increase was markedly reduced following teriparatide and high-dose HBA treatments (*p* < 0.05). BALP levels were significantly reduced in the osteoporosis group compared to the healthy control group, and this reduction was significantly prevented by high-dose lentinan treatment (*p* < 0.05). Although high-dose HBA treatment numerically attenuated the reduction in BALP levels, no statistically significant differences were found compared to the control and teriparatide groups (*p* > 0.05).

**FIGURE 1:**
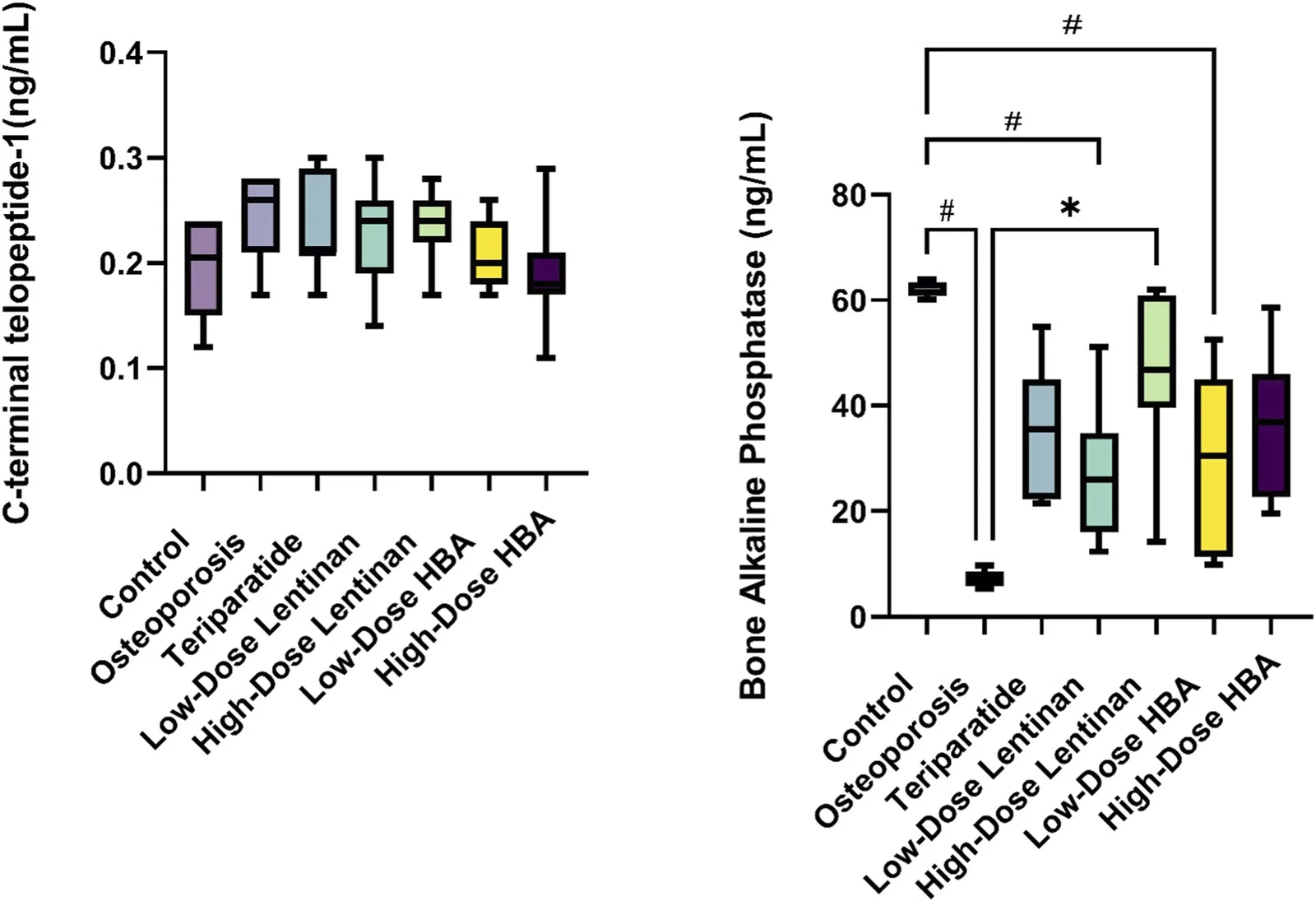
Effects of hydroxybenzoic acid (50–100 μg/kg) and lentinan (200–400 mg/kg) administration on c-terminal telopeptide of type i collagen (CTX-1) and bone alkaline phosphatase (BALP) parameters in mice with an experimental osteoporosis model (median). Effects of p-hydroxybenzoic acid (50–100 μg/kg) and lentinan (200–400 mg/kg) on serum C-terminal telopeptide of type I collagen (CTX-1) and bone-specific alkaline phosphatase (BALP) in mice with experimentally induced glucocorticoid-induced osteoporosis. Data are presented as median with interquartile range (box) and minimum–maximum values (whiskers). n = 8 (healthy control) and n = 10 (all other groups). Group comparisons were performed by Kruskal–Wallis test followed by Dunn–Bonferroni *post hoc* test. *p < 0.05 versus the osteoporosis group; ^#^p < 0.05 versus the healthy control group. No significant differences among groups were detected for CTX-1. HBA, p-hydroxybenzoic acid.

**FIGURE 2:**
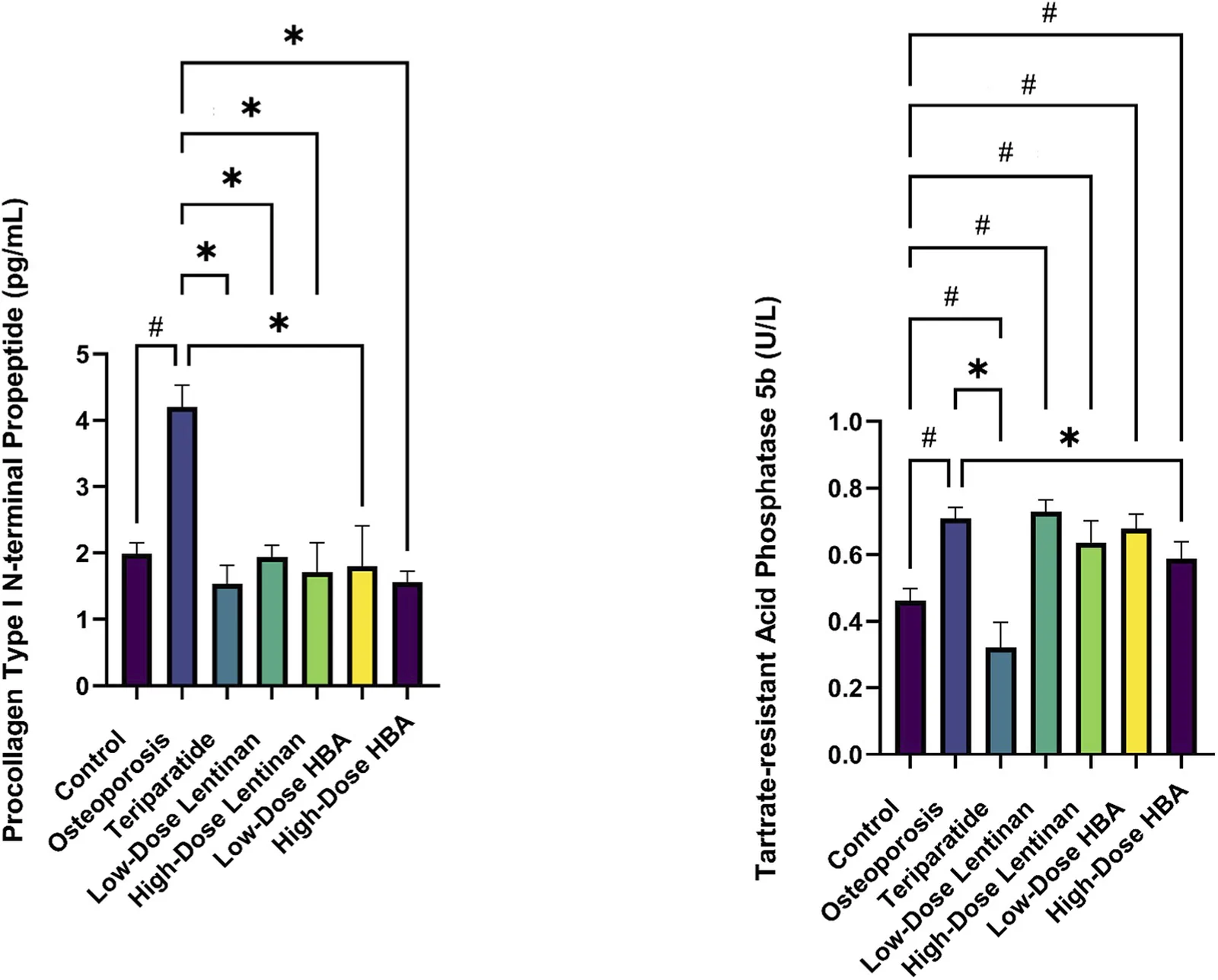
Effects of hydroxybenzoic acid (50–100 μg/kg) and lentinan (200–400 mg/kg) Administration on procollagen type I N-terminal propeptide (PINP) and tartrate-resistant acid phosphatase 5b (TRACP-5b) parameters in mice with an experimental osteoporosis model (mean). Effects of p-hydroxybenzoic acid (50–100 μg/kg) and lentinan (200–400 mg/kg) on serum procollagen type I N-terminal propeptide (PINP) and tartrate-resistant acid phosphatase 5b (TRACP-5b) in mice with experimentally induced glucocorticoid-induced osteoporosis. Data are presented as mean ± standard deviation. n = 8 (Healthy Control) and n = 10 (all other groups). Group comparisons were performed by one-way analysis of variance followed by Tukey’s honestly significant difference *post hoc* test. *p < 0.05 versus the osteoporosis group; ^#^p < 0.05 versus the healthy control group. HBA, p-hydroxybenzoic acid.

### 3.2 Hematological findings

The hematological parameters of the study groups are presented in Table 2. HGB and HCT levels were significantly lower in the high-dose lentinan group compared with the healthy control group (*p* < 0.05). HCT levels were also significantly lower in the low-dose lentinan group compared with the healthy control group (*p* < 0.05). WBC levels were significantly higher in the low- and high-dose lentinan groups, compared with the healthy control group (*p* < 0.05). A statistically significant increase in PLT levels was observed in the high-dose HBA group compared with the osteoporosis group (*p* < 0.05).

**TABLE 2:** Effects of hydroxybenzoic acid (50–100 μg/kg) and lentinan (200–400 mg/kg) administration on hematological parameters in mice with an experimental osteoporosis model (mean ± SEM).

Parameters/groups	Control	Osteoporosis	Teriparatide	Low-dose lentinan	High-dose lentinan	Low-dose HBA	High-dose HBA
WBC (10^3^/µL)	5.58 ± 0.70	9.57 ± 1.77	7.57 ± 0.80	10.19 ± 0.20^#^	10.60 ± 1.48^#^	6.11 ± 0.10	5.57 ± 0.40
RBC (10^6^/µL)	8.63 ± 0.16	8.59 ± 0.16	8.98 ± 0.17	8.22 ± 0.20	8.27 ± 0.21	8.65 ± 0.19	8.65 ± 0.11
HGB (g/dL)	13.80 ± 0.19	13.31 ± 0.17	14.05 ± 0.35	12.78 ± 0.20	12.46 ± 0.43^#^	13.28 ± 0.29	13.90 ± 0.22
HCT (%)	44.68 ± 0.65	43.33 ± 0.35	43.40 ± 1.01	40.66 ± 0.64^#^	40.71 ± 1.00^#^	42.98 ± 0.53	44.00 ± 0.74
PLT (10^3^/µL)	1165.80 ± 55.0	1022.0 ± 9.5	877.33 ± 141.4	1248.40 ± 50.6	1255.83 ± 32.3	1090.50 ± 54.1	1378.33 ± 61.4*

WBC, white blood cells; RBC, red blood cells; HGB, hemoglobin; HCT, hematocrit; PLT, platelets.

* Indicates significant differences compared to the Osteoporosis group.

^#^ Indicates significant differences compared to the Healthy Control group.

### 3.3 Histopathological changes in bone tissue

Histopathological and immunohistochemical evaluation results are presented in Table 3. Histopathological examination revealed that the control groups exhibited no pathological lesions, displaying a normal histological appearance characterized by intact Haversian systems, classical compact bone covered externally with periosteum, and bone marrow centrally located within the medullary cavity. In the osteoporosis group, thinning of the bone trabeculae due to resorption and loss of basophilia in the bone matrix due to demineralization were observed, accompanied by the formation of osteoporotic cavities and fractures. Additionally, broad refractile areas indicating degenerative and necrotic changes were detected within the osteoid tissue. More prominent erosive lesions were present in the periosteum and endosteum, along with enlargement of subperiosteal spaces. Erosive alterations in compact bone were also associated with irregular cellular columns, disorganized bone network formation, and widespread cement line structures. Enlarged lacunae were occasionally empty or contained degenerative osteocytes. In the other treatment groups, compared with the osteoporosis group, the severity of degenerative and necrotic lesions as well as osteoporotic destructive changes was reduced in a dose- and treatment-dependent manner, with the following order of improvement: low-dose lentinan, low-dose HBA, high-dose lentinan, high-dose HBA, and teriparatide. These changes were accompanied by restorative alterations approaching the appearance of the healthy control group (Figure 3).

**TABLE 3:** Histopathological and immunohistochemical scoring results of the femur bone in mice with an experimental osteoporosis model treated with p-hydroxybenzoic acid and lentinan (median (IQR)).

Parameters/groups	Control	Osteoporosis	Teriparatide	Low-dose lentinan	High-dose lentinan	Low-dose HBA	High-dose HBA
ORD	0 (0)	3.0 (1.0)^#^	0 (0.25)*	2.5 (1.0)^#^	1.5 (1.25)	2.0 (1.25)^#^	0.5 (2.0)
DNL	0 (0)	3.0 (0.25)^#^	0 (1.0)*	2.5 (1.0)^#^	1.5 (1.25)	2.0 (1.25)^#^	0.5 (2.0)
CTT	0 (0)	3.0 (0.25)^#^	0 (0.25)*	3.0 (1.0)^#^	1.5 (1.25)	2.0 (1.25)	0.5 (2.0)
TNF-α	0 (0)	3.0 (0.25)^#^	0 (0.25)*	2.0 (1.0)^#^	1.5 (1.0)	2.0 (0.5)	0.5 (1.25)*
IL-1β	0 (0)	3.0 (0.25)^#^	0 (0.25)*	2.0 (1.0)^#^	1.0 (1.0)	2.0 (1.0)	0.5 (1.25)*
HSP-27	0 (0)	3.0 (0.25)^#^	0 (1.0)*	2.0 (1.0)^#^	1.5 (1.0)	2.0 (1.25)	0.5 (1.25)*
RANK	0 (0)	3.0 (1.0)^#^	0 (0.25)*	2.0 (1.25)^#^	1.0 (1.0)	1.5 (1.25)	0.5 (1.0)*
RANKL	0 (0)	2.5 (1.0)^#^	0 (0.25)*	2.0 (0.50)^#^	1.5 (1.0)	2.0 (1.25)^#^	0.5 (1.25)

ORD, Osteoporotic resorption-demineralization; DNL, Degenerative-necrotic lesions; CTT, cortical and trabecular thinning; TNF-α, Tumor necrosis factor-α; IL-1β, Interleukin-1β; HSP-27, Heat shock protein-27; RANK, receptor activator of nuclear factor kappa; RANKL, Receptor activator of nuclear factor kappa-B, ligand; HBA, p-Hydroxybenzoic acid.

* Indicates significant changes compared to the osteoporosis group.

^#^ Indicates significant changes compared to the healthy control group.

**FIGURE 3:**
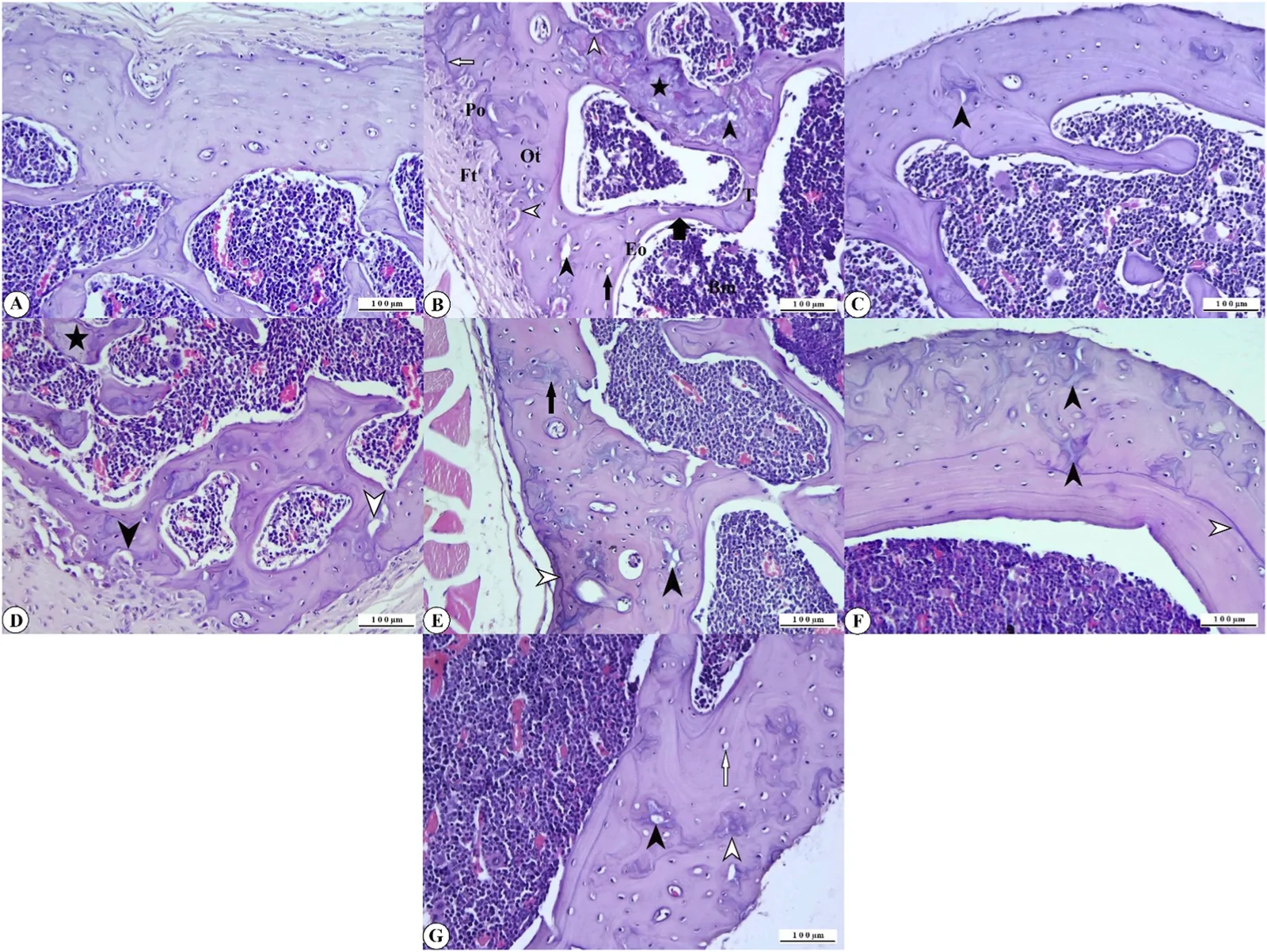
Effects of hydroxybenzoic acid (50–100 μg/kg) and lentinan (200–400 mg/kg) administration on histopathological findings in mice with an experimental osteoporosis model (HE, ×200, Bar = 100 µm). Histopathological examination revealed that the control groups exhibited no pathological lesions, displaying a normal histological appearance characterized by an intact Haversian system, classical compact bone covered externally by periosteum, and bone marrow located in the central region. In the osteoporosis group, thinning of bone trabeculae due to resorption and loss of basophilia in the bone matrix caused by demineralization were observed, accompanied by osteoporotic cavities and fissures. Extensive refractile areas indicative of degenerative and necrotic changes in the osteoid tissue were noted. More pronounced erosive wear was evident in the periosteum and endosteum, along with widening of the subperiosteal spaces. Erosive changes in the compact bone were also associated with irregular cell columns, disorganized bone meshwork, and the formation of extensive cement lines. Enlarged lacunae were occasionally empty or contained degenerative osteocytes. In the other treatment groups, compared to the MP group, the severity of degenerative and necrotic lesions and osteoporosis-related destructive changes decreased in the following order: low-dose lentinan, low-dose hydroxybenzoic acid, high-dose lentinan, high-dose hydroxybenzoic acid, and teriparatide. Instead, histological changes resembling restorative features similar to those of the control group were observed (Figure 3). **(A)** Control group: normal histological appearance of bone tissue. **(B)** Osteoporosis group: refractile areas indicative of degenerative and necrotic changes in the osteoid tissue (asterisk), osteoporotic cavities and ruptures (arrowheads), thinning of trabeculae (thick arrow), enlarged lacunae containing degenerative osteocytes (arrow), resolutive erosion in the endosteum and periosteum (open arrowheads), and deep cement lines in the osteoid tissue (open arrows). Po, periosteum; Ot, osteoid tissue; Ft, fibrous tissue; Eo, endosteum; T, trabeculae; Bm, bone marrow. **(C)** Teriparatid group: osteoporotic cavities and mild resorption areas (arrowhead). **(D)** Low- dose lentinan group: refractile areas indicative of degenerative and necrotic changes in the osteoid tissue (asterisk), osteoporotic cavities and ruptures (open arrowhead), subperiosteal erosion (arrowhead). **(E)** Low-dose hydroxybenzoic acid group: refractile areas indicative of degenerative and necrotic changes in the osteoid tissue (arrow), osteoporotic cavities (arrowhead), and deep cement lines in the osteoid tissue (open arrow). **(F)** High-dose lentinan group: refractile areas indicative of degenerative and necrotic changes in the osteoid tissue (arrow), and deep cement lines in the osteoid tissue (open arrow). **(G)** High-dose hydroxybenzoic acid group: refractile areas indicative of degenerative and necrotic changes in the osteoid tissue (arrow), osteoporotic cavities (arrowhead), and lacunae with degenerative osteocytes (open arrow).

### 3.4 Immunohistochemical assessment of inflammatory, oxidative stress, and bone resorption markers

Immunohistochemical analysis was performed to assess inflammatory, oxidative stress, and bone resorption markers in bone tissue. For the evaluation of inflammation, the widely used biomarkers TNF-α and IL-1β were examined. No TNF-α or IL-1β immunopositivity was detected in the bone tissue of the control groups. In contrast, the osteoporosis group exhibited strong TNF-α and IL-1β expression. Among the treatment groups, TNF-α and IL-1β expression levels were reduced compared to the osteoporosis group, with the degree of reduction following the order: teriparatide, low-dose lentinan, high-dose lentinan, low-dose HBA, and high-dose HBA (Figure 4).

**FIGURE 4:**
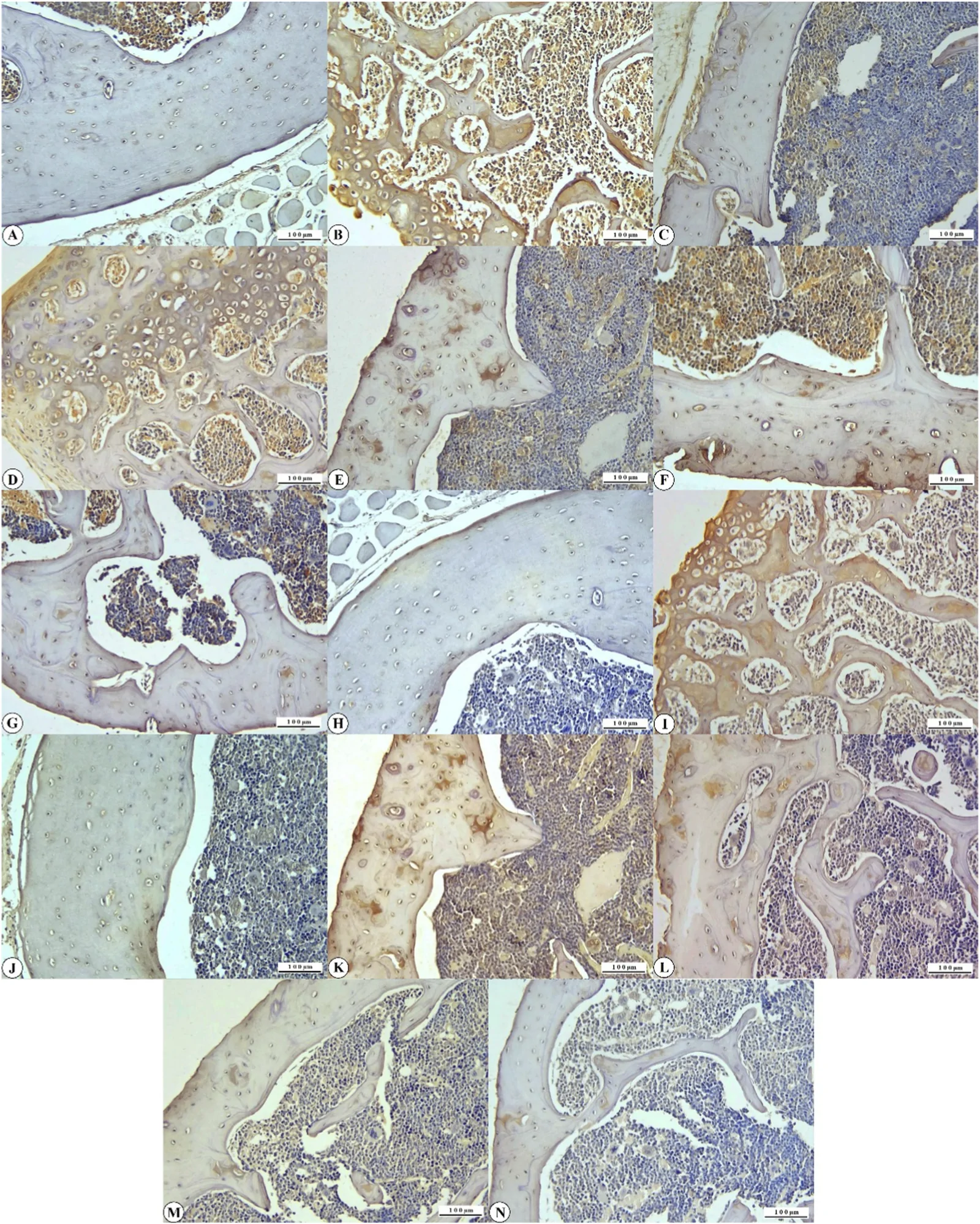
Immunohistochemical changes associated with inflammation (TNF-α and IL-1β) in experimental osteoporosis mice following administration of hydroxybenzoic acid (50–100 μg/kg) and lentinan (200–400 mg/kg) (IHC, ×200, Bar = 100 µm). Immunohistochemical analysis was performed to evaluate inflammation in bone tissue by assessing the expression of widely used and current biomarkers TNF-α and IL-1β. No TNF-α immunopositivity was detected in the bone tissues of control groups. In contrast, the osteoporosis group exhibited intense TNF-α expression. The expression levels of TNF-α were reduced in the low-dose lentinan, low-dose hydroxybenzoic acid, high-dose lentinan, high-dose hydroxybenzoic acid, and teriparatide groups, respectively, compared to the osteoporosis group. Similarly, IL-1β immunopositivity was absent in the control group but was markedly increased in the osteoporosis group. A stepwise decrease in IL-1β expression was observed in the low-dose lentinan, low-dose hydroxybenzoic acid, high-dose lentinan, high-dose hydroxybenzoic acid, and teriparatide groups compared to the osteoporosis group (Figure 4). In the control group, bone tissue showed no immunopositivity for TNF-α **(A)** and IL-1β **(H)**. In contrast, the osteoporosis group exhibited intense expression of both TNF-α **(B)** and IL-1β **(I)** in bone tissue. The teriparatide group demonstrated mild expression of TNF-α **(C)** and IL-1β **(J)**. TNF-α and IL-1β expressions were also observed in the low-dose lentinan **(D, K)**, low-dose hydroxybenzoic acid **(E, L)**, high-dose lentinan **(F, M)**, and high-dose hydroxybenzoic acid **(G, N)** groups, with varying intensities (Figure 4).

Oxidative stress in bone tissue was evaluated by HSP-27 expression. No HSP-27 immunopositivity was observed in the control groups, whereas strong HSP-27 expression was detected in the osteoporosis group. Similar to the inflammatory markers, HSP-27 expression was reduced in the treatment groups compared to the osteoporosis group, with the same order of reduction: teriparatide, low-dose lentinan, high-dose lentinan, low-dose HBA, and high-dose HBA (Figure 5).

**FIGURE 5:**
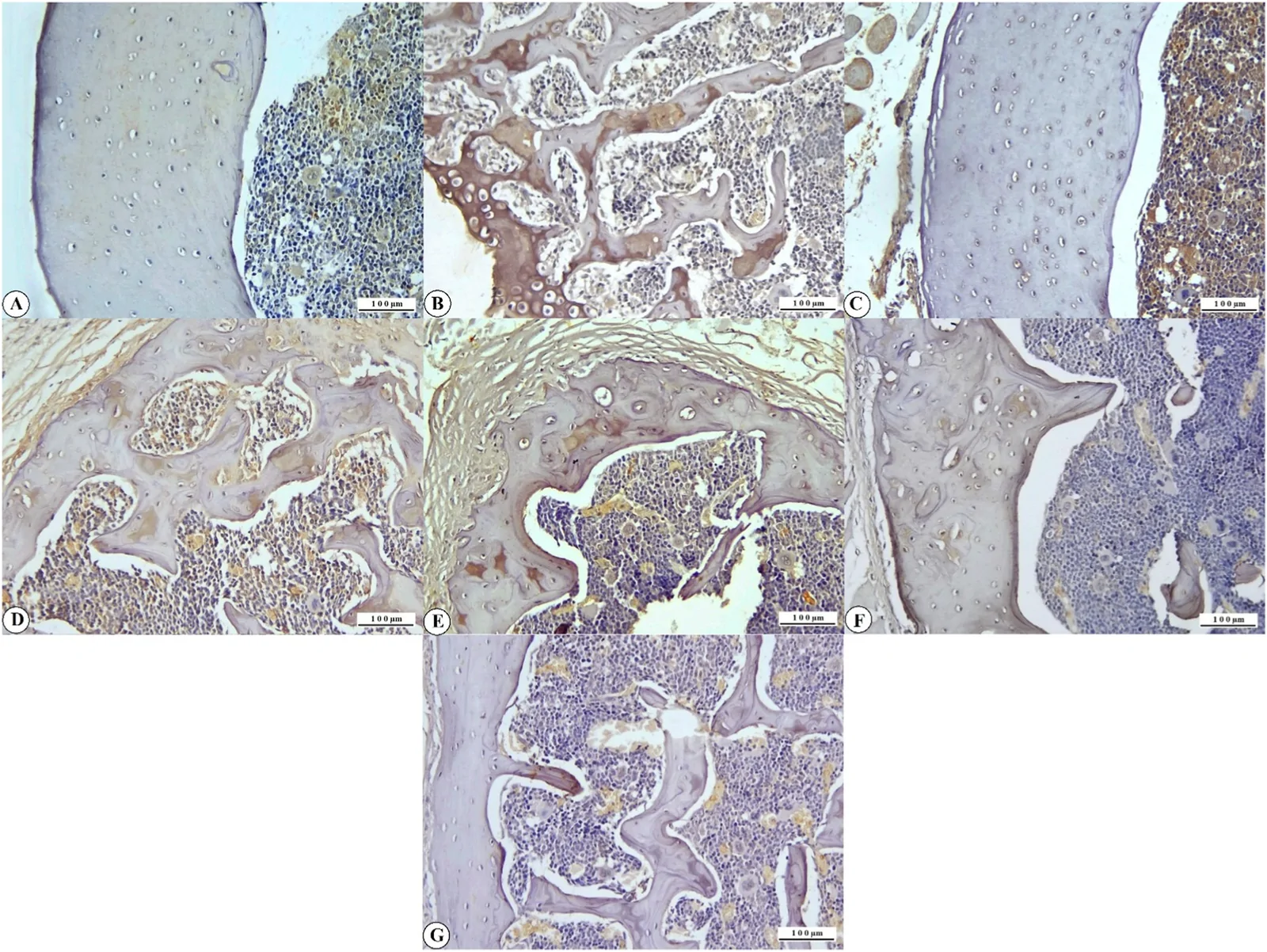
Immunohistochemical changes associated with oxidative stress (HSP-27) in experimental osteoporosis mice following administration of hydroxybenzoic acid (50–100 μg/kg) and lentinan (200–400 mg/kg) (IHC, ×200, Bar = 100 µm). Oxidative stress in bone tissue was evaluated by assessing HSP-27 expression. No HSP-27 immunopositivity was detected in the control groups, whereas intense expression was observed in the osteoporosis group. Subsequent groups—low-dose lentinan, low-dose hydroxybenzoic acid, high-dose lentinan, high-dose hydroxybenzoic acid, and teriparatide—showed progressively reduced HSP-27 expression relative to the osteoporosis group (Figure 5). In the control group, bone tissue showed no immunopositivity for the oxidative stress biomarker HSP-27 **(A)**. In contrast, the osteoporosis group exhibited intense HSP-27 expression in bone tissue **(B)**. The teriparatide group demonstrated mild HSP-27 expression **(C)**. HSP-27 expression was also observed in the low-dose lentinan **(D)**, low-dose hydroxybenzoic acid **(E)**, high-dose lentinan **(F)**, and high-dose hydroxybenzoic acid **(G)** groups, with varying intensities (Figure 5).

Bone resorption activity was assessed by examining the expression of RANK and RANKL, which play pivotal roles in the regulation of bone remodeling. Control groups exhibited no RANK or RANKL immunopositivity, while the osteoporosis group showed strong expression of both markers. In the treatment groups, RANK and RANKL expressions were decreased compared to the osteoporosis group, with the reduction pattern again following the sequence: teriparatide, low-dose lentinan, high-dose lentinan, low-dose HBA, and high-dose HBA (Figure 6).

**FIGURE 6:**
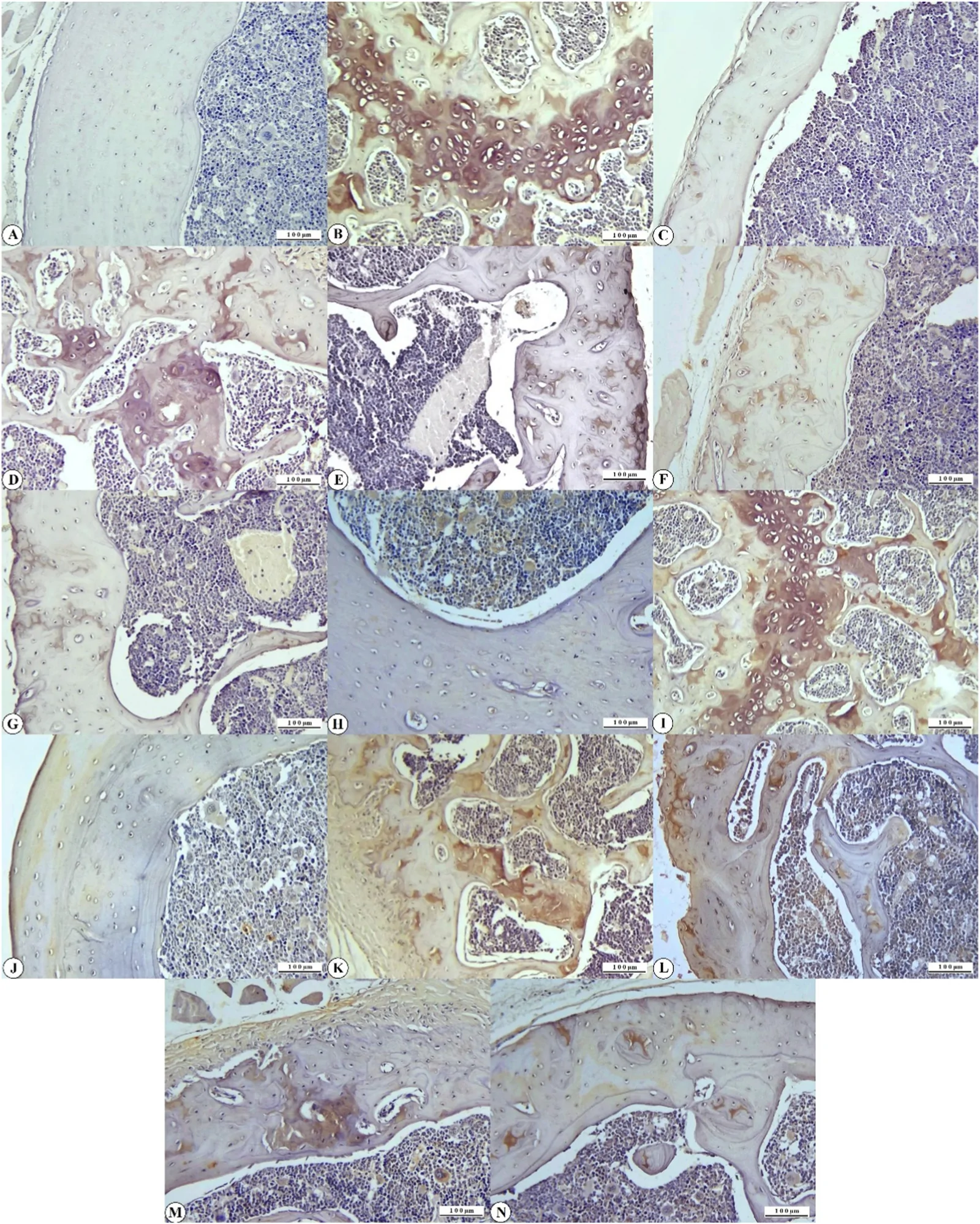
Immunohistochemical Changes Associated with osteoid biomarkers (RANK and RANKL) in Experimental Osteoporosis Mice Following Administration of Hydroxybenzoic Acid (50–100 μg/kg) and Lentinan (200–400 mg/kg) (IHC, ×200, Bar = 100 µm). Immunohistochemical evaluation of RANK and RANKL expression in experimental groups. RANK immunopositivity was absent in the control groups but markedly increased in the osteoporosis group. In contrast, low- and high-dose lentinan, low- and high-dose p-hydroxybenzoic acid, and teriparatide treatments attenuated RANK expression compared with the osteoporosis group. Similarly, RANKL immunopositivity was undetectable in the control group but strongly expressed in the osteoporosis group, with a stepwise reduction observed following all treatment interventions (Figure 6). In the control group, bone tissue showed no immunopositivity for RANK **(A)** and RANKL **(H)**. In contrast, the osteoporosis group exhibited intense expression of both RANK **(B)** and RANKL **(I)** in bone tissue. The teriparatide group demonstrated mild RANK **(C)** and RANKL **(J)** expression. RANK and RANKL expressions were also observed in the low-dose lentinan **(D, K)**, low-dose hydroxybenzoic acid **(E, L)**, high-dose lentinan **(F, M)**, and high-dose hydroxybenzoic acid **(G, N)** groups, with varying intensities (Figure 6).

## 4 Discussion

Glucocorticoids, when used in high doses and for prolonged periods, increase bone resorption, leading to decreased bone mineral density and an elevated risk of fractures, while simultaneously suppressing osteoblastogenesis and promoting osteoclastic activity (Compston, 2010; Canalis, 2005). The findings of our study demonstrate that especially at high doses, administration of HBA and lentinan significantly reduces glucocorticoid-induced bone loss both biochemically and histopathologically. These results suggest that HBA and lentinan, through distinct mechanisms, each contributed to the preservation of bone tissue (Manuja et al., 2013; Safarova, 2022; Lee H. J. et al., 2020). Their combined use was not tested in the present study.

Osteoporosis is a multifactorial disorder characterized by reduced bone mineral density and deterioration of bone microarchitecture, with vitamin D deficiency being a key etiological factor. Prolonged glucocorticoid administration impairs intestinal vitamin D absorption and suppresses renal 1α-hydroxylase activity, thereby reducing circulating 1,25(OH)_2_D_3_ levels, which accelerates bone resorption through decreased calcium absorption and compensatory parathyroid hormone secretion (Akeno et al., 2000; Feger et al., 2021). In the present study, high-dose lentinan and both low- and high-dose p-hydroxybenzoic acid (HBA) effectively prevented glucocorticoid-induced vitamin D depletion. Although literature on lentinan’s direct role in vitamin D metabolism is limited, previous work has shown that lentinan reduces serum osteocalcin and IL-6 while preserving bone mineral density (Lee H. J. et al., 2020), and that *L. edodes* extracts enhance osteoblast mineralization and alkaline phosphatase (ALP) activity (Saif et al., 2013). These findings suggest that lentinan may indirectly support vitamin D homeostasis via osteogenic promotion. HBA, with its potent antioxidant and estrogen-mimicking properties, may counteract glucocorticoid-induced oxidative stress and estrogen depletion, thereby stabilizing vitamin D metabolism and bone turnover (López-Herrador et al., 2025; Nguyen et al., 2022).

Glucocorticoids are known to downregulate insulin-like growth factor 1 (IGF-1) production in osteoblasts, impairing bone formation (Ilias et al., 2022; Lin et al., 2014; Smink et al., 2003). IGF-1 activation of the PI3K/AKT pathway mitigates glucocorticoid-induced inhibition of osteoblast differentiation (Jiang et al., 2019). Lentinan-rich *L. edodes* extracts have been reported to suppress RANKL-induced osteoclastogenesis by downregulating NFATc1 and inflammatory cytokines, thereby reversing glucocorticoid-induced bone loss (Lee H. et al., 2020; Lindequist, 2024). Similarly, phenolic derivatives of HBA, including syringic and protocatechuic acids, exert anti-osteoclastogenic effects via downregulation of the RANKL signaling pathway and activation of Nrf2, while enhancing osteoblast differentiation (Saleh et al., 2022; Tanaka et al., 2017; Wu et al., 2016). In this study, high-dose HBA significantly reduced TRACP-5b levels, suggesting a pronounced anti-resorptive profile, whereas lentinan’s effect on this parameter may be limited depending on factors such as dose, duration of administration, or model specificity.

Glucocorticoid exposure rapidly suppresses serum markers of bone formation such as osteocalcin, ALP (Weinstein et al., 1998). While limited research exists on the effects of lentinan and HBA on these markers, shiitake mushroom extracts have been shown to enhance type I collagen synthesis, ALP activity, and mineralization (Lee H. J. et al., 2020; Saif et al., 2013), and to reduce abnormally elevated osteocalcin levels in ovariectomized rat models. In the present study, high-dose lentinan increased bone-specific ALP and serum phosphorus levels but decreased P1NP, suggesting enhanced osteoblast maturation and a potential shift from early matrix synthesis toward mineralization. Conversely, high-dose HBA reduced both TRACP-5b and P1NP levels, indicating suppression of bone resorption alongside limited matrix synthesis, consistent with a predominantly anti-resorptive mechanism. These findings highlight the distinct but complementary roles of lentinan and HBA in preserving bone homeostasis under glucocorticoid-induced osteoporotic conditions.

In the present study, histopathological and immunohistochemical analyses revealed that the glucocorticoid-induced osteoporosis group exhibited significant bone resorption and microstructural deterioration compared to the healthy control group. Specifically, osteoclastic bone destruction and demineralization (ORD), degenerative-necrotic lesions (DNL), and cortical/trabecular thinning (CTT) scores were significantly elevated in femur samples of osteoporotic mice; concomitantly, the expression of pro-inflammatory and resorptive mediators such as TNF-α and IL-1β was increased. Additionally, HSP-27, an oxidative stress marker, along with RANK and RANKL—indicators of osteoclastic activity and bone resorption—were elevated following glucocorticoid administration. In a dexamethasone-induced osteoporosis model, significant reductions in trabecular bone thickness, increased resorption, shrinkage of cartilage nuclei, and decreased chondrocyte counts in bone marrow spaces were observed (Abdelfattah et al., 2023). Glucocorticoids enhance TRAP-5b and RANKL expression in stromal and osteoblastic cells, thereby promoting osteoclastogenesis and prolonging osteoclast lifespan. Furthermore, evidence indicates that glucocorticoids suppress osteoblastogenesis, impair osteoblastic differentiation and maturation, and reduce both the number and function of osteoblasts (Canalis et al., 2007; Hofbauer et al., 2009; Piemontese et al., 2016). Prednisolone administration contributes to the activation of the RANK/NF-κB axis in bone marrow cells and increases the circulating pre-osteoclast pool (Shymanskyi et al., 2018). The synergistic effect of HSP27 with RANKL in promoting monocyte migration and thereby osteoclast formation represents a potential mechanism accelerating bone resorption in osteoporosis (Daswani et al., 2015). Glucocorticoid exposure induces osteocyte apoptosis and increases the release of pro-inflammatory cytokines such as TNF-α and IL-1β, which stimulate osteoclastogenesis and accelerate bone resorption. The destructive effects of TNF-α on osteocytes and osteoblasts disrupt cellular homeostasis in bone remodeling, leading to structural deterioration in glucocorticoid-induced osteoporosis (Mazziotti et al., 2007; Wang et al., 2019).

4-HBA demonstrates significant anti-inflammatory effects by markedly reducing TNF-α levels (Yoo et al., 2017), suggesting it may indirectly modulate inflammation-associated pathways such as IL-1β and RANK/RANKL signaling (Florescu et al., 2023). Lentinan suppresses TNF-α-mediated inflammation by targeting inflammatory processes, reducing the expression of the cell surface TNF receptor TNFR1, and inhibiting the NF-κB signaling pathway. This mechanism enables lentinan to act as a potent anti-inflammatory agent by limiting pro-inflammatory cytokine production (Liu et al., 2019; Nishitani et al., 2013). However, literature searches have yet to provide direct evidence of specific interactions between 4-HBA or lentinan and HSP-27, RANK, or RANKL. In the current study, increased oxidative stress and inflammatory cytokines in the osteoporosis group phosphorylated and activated HSP-27, and the elevated pHSP27 likely enhanced osteoclast precursor migration to the bone microenvironment and osteoclastic activity via activation of the RANK/RANKL pathway, thereby inducing osteoporosis. The administered treatments markedly reduced bone tissue damage and inflammatory response, as observed histopathologically and immunohistochemically.

Particularly, teriparatide, an anabolic agent, demonstrated the expected reparative effects in osteoporotic bone by nearly normalizing histopathological scores and significantly reducing the immunohistochemical expression of markers such as TNF-α, IL-1β, RANK, and RANKL. High-dose lentinan, through its immunomodulatory and anti-inflammatory properties, suppressed the NF-κB pathway, reducing the overexpression of inflammatory cytokines including TNF-α and IL-1β, and alleviated bone resorption and thinning lesions, thus preventing osteoporosis. Nevertheless, high-dose lentinan treatment yielded only partial improvement, producing similar results in both healthy control and osteoporotic groups. Notably, the group treated with high-dose 4-HBA showed significantly decreased histopathological damage scores and levels of immunohistochemical markers TNF-α, IL-1β, HSP-27, RANK, and RANKL compared to the osteoporosis group. The bone-protective effect of 4-HBA was associated with reduced RANK and RANKL expression and decreased inflammatory cytokine levels. These effects may be related, at least in part, to the previously reported antioxidant and estrogen-like properties of 4-HBA.

The present findings indicate that teriparatide, lentinan and p-HBA act on different arms of bone remodelling and should therefore be regarded as complementary rather than competing interventions. Teriparatide, as expected of an anabolic agent, produced the most complete normalisation of histopathological scores and of all immunohistochemical markers examined. Lentinan acted predominantly on the osteoblastic and anti-inflammatory side, as reflected by the preservation of bone-specific alkaline phosphatase and serum phosphorus and by the reduction of TNF-α and IL-1β immunoreactivity, whereas its effect on the resorptive marker TRACP-5b was limited. High-dose p-HBA showed the opposite profile, with a pronounced anti-resorptive signature—reduced TRACP-5b together with decreased RANK, TNF-α, IL-1β and HSP-27 immunoreactivity—accompanied by a decrease rather than an increase in PINP, indicating suppression of bone turnover rather than stimulation of matrix synthesis. This mechanistic dissociation suggests that the two compounds address different components of glucocorticoid-induced bone loss and that their combination, which was not tested here, merits investigation. From a translational perspective, neither p-HBA nor lentinan has an approved indication in osteoporosis, and the present evidence is preclinical, obtained in male mice over a 10-week period and without densitometric or biomechanical confirmation. Therefore, these agents should be regarded as potential adjunctive therapies rather than substitutes for established anti-osteoporotic treatments in patients receiving long-term glucocorticoid therapy. Current standard-of-care management, including adequate calcium and vitamin D supplementation together with an approved antiresorptive or anabolic agent when indicated, should remain the cornerstone of treatment. Further studies incorporating dose–response and combination designs, together with micro-CT and biomechanical assessments, are warranted to validate the present findings and to establish their potential clinical relevance.

Haematological parameters generally remained within the reference intervals reported for albino mouse stocks and inbred albino strains (Serfilippi et al., 2003; O'Connell et al., 2015), with only a modest increase in total leukocyte count observed in the lentinan-treated groups. Given the well-documented immunomodulatory activity of lentinan, this finding may reflect its pharmacodynamic effects rather than systemic toxicity. Overall, the observed variations in blood urea nitrogen (BUN), creatinine, and haematological parameters remained within the reported reference ranges, with no evidence of clinically relevant hepatic, renal, or haematological toxicity associated with lentinan or 4-HBA administration during the 10-week treatment period (Coman et al., 2018; IDEXX Laboratories, 2024).

## 5 Conclusion

This study demonstrates that HBA and lentinan possess therapeutic potential against glucocorticoid-induced osteoporosis by protecting bone metabolism through multiple mechanisms. High-dose HBA attenuated glucocorticoid-induced decreases in 25(OH)D_3_ and IGF-1 levels and reduced osteoclastogenesis, as evidenced by the suppression of RANKL–RANK–NFκB pathway-related markers. These effects may be associated with the previously reported estrogen-like and antioxidant properties of HBA; however, these mechanisms were not directly investigated in the present study. Lentinan, through its immunomodulatory and osteoblast-stimulating effects, attenuated pro-inflammatory cytokine expression and promoted bone formation. Histopathological analyses confirmed preservation of trabecular integrity and reduction of osteoporotic lesions in treated groups.

Overall, it can be concluded that HBA primarily exerts its effects by reducing osteoclastic activity, whereas lentinan predominantly promotes osteoblastic activity. In several histopathological and immunohistochemical parameters, high-dose HBA approached but did not exceed—the effect of teriparatide, whereas the effect of lentinan was more limited. These findings identify HBA and lentinan as candidate adjuncts to, rather than substitutes for, established anti-osteoporotic therapy during long-term glucocorticoid treatment, and indicate that their efficacy and safety require confirmation in further preclinical and, subsequently, clinical studies. Further research is warranted to clarify their pharmacokinetics, clinical efficacy, and potential pharmaceutical applications.

## 6 Limitations

One limitation of the present study is the absence of pharmacokinetic characterization and a formal dose–response assessment for p-HBA and lentinan in the experimental model. Although the doses used in the present study were selected based on previously reported *in vivo* evidence of biological activity and tolerability, comprehensive dose optimization and pharmacokinetic evaluation are warranted to establish the optimal therapeutic range and to facilitate the translation of these findings into clinical applications.

Another limitation of the present study is that RANK and RANKL expression was assessed by immunohistochemistry without complementary quantitative validation by Western blot or ELISA. Although immunohistochemistry provided valuable information regarding the tissue localization and distribution of these proteins, future studies incorporating quantitative protein-based approaches are warranted to further substantiate the involvement of the RANK/RANKL pathway in the observed effects.

## Data Availability

The original contributions presented in this study are included in the article. Further inquiries can be directed to the first author.
